# Phenotypical and genotypical resistance testing of *Pasteurella multocida* isolated from different animal species in Austria

**DOI:** 10.3389/fvets.2025.1640536

**Published:** 2025-08-13

**Authors:** Leonard Barta, Anna Stöger, Daniel Polzer, Werner Ruppitsch, Friedrich Schmoll, Tatjana Sattler

**Affiliations:** ^1^Institute for Veterinary Investigations Mödling, Austrian Agency for Health and Food Safety, Mödling, Austria; ^2^Institute of Medical Microbiology and Hygiene, Austrian Agency for Health and Food Safety, Vienna, Austria; ^3^Institute of Hygiene and Medical Microbiology, Medical University Innsbruck, Innsbruck, Austria; ^4^University of Leipzig, Clinic for Ruminants and Swine, Leipzig, Germany

**Keywords:** bacteriology, antimicrobial resistance, genotype, phenotype, antimicrobial susceptibility testing

## Abstract

**Introduction:**

*Pasteurella multocida* is an economically important pathogen in veterinary medicine. Data on its antimicrobial resistance vary widely across regions. Furthermore, most of the found literature focuses on phenotypic resistance testing. To date, no study has examined *P. multocida* resistance in Austria, and no national surveillance program exists.

**Methods:**

In this study, we tested 276 isolates of *P. multocida* from different hosts including farm animals, pets, wildlife and humans. Susceptibility testing was performed using three different variants of the broth microdilution method against 16 antibiotics, applying veterinary specific breakpoints referenced from CLSI: the CAMHB method using cation adjusted Mueller Hinton Broth, the LHB method supplemented with laked horse blood and the LHB + CO_2_ method, which additionally included an enriched CO_2_ atmosphere. Whole genome sequencing was then performed to identify resistance genes. Genomic data and the results from the phenotypical resistance testing were compared to determine the most suitable method for the detection of resistance.

**Results:**

About 20% of bovine isolates and 9% of pig isolates carried at least one resistance gene. No resistance genes were detected in isolates from other hosts. The most commonly detected resistance genes were against tetracyclines, aminoglycosides and sulphonamides. Resistance against florfenicol and macrolides was scarce and only present in bovines. Three or more different resistance genes were found in 3% of porcine strains and 10% of cattle strains. In pig isolates, the comparison of phenotype and genotype revealed a good concordance rate using both the CAMHB and LHB methods. Method LHB + CO_2_ yielded major discrepancies in macrolide susceptibility results. In cattle, CAMHB method showed a high concordance, however, it failed to identify resistant isolates. While the LHB and LHB + CO_2_ methods demonstrated effective detection of resistance genes, they were associated with a higher rate of false-positive results for ampicillin resistance.

**Discussion:**

We recommend performing antimicrobial resistance testing of *P. multocida* with the supplementation of LHB. Despite the occurrence of false positive results, it is still the most suitable method to detect resistance genes. Our results suggest good efficacy of antibiotics against *P. multocida* in Austria, however, the risk posed by strains carrying multiple resistance genes should not be overlooked.

## 1 Introduction

Antimicrobial resistance (AMR) is currently considered as one of the top 10 threats to humanity according to the World Health Organization (1). Many antibiotics that were able to treat severe infectious diseases in humans and animals have lost their capability to kill or inhibit the respective bacterial agent (2, 3). Despite the great efforts which have been made in a One Health aspect to tackle this serious problem, the global use of antimicrobials is still on the rise (4). For a better understanding of AMR it is important to know that this phenomenon is not new. A study by Olaitan and Rolain demonstrated that antimicrobial resistance genes were present even before mankind started using antibiotics (5). Resistance against commonly used antibiotics in veterinary medicine, such as *β*-lactams, macrolides or amphenicols, has been dated to four million years ago (6). The long history of the bacterial resistome is an impressive example of the ability of bacteria to adapt to new circumstances and to overcome threats. It highlights the everlasting battle between bacterial survival and their challenge agents like antibiotics.

Apart from the natural development of AMR in bacteria, which plays a subordinate role, the recent threat is human made (7). Implemented by the European Union, Austria has to monitor and record its veterinary antibiotic consumption as well as the antimicrobial resistance in zoonotic bacteria and commensal bacteria of animal origin (8). Although these results, presented in the annual Austrian resistance report (AURES) provide important data, the platform is not a useful tool for practitioners when it comes to the treatment of infected animals (9). Unfortunately, in Austria there are currently no reports on antimicrobial resistance in animal pathogens. In many countries like Germany (10), United Kingdom (11), Czech Republic (12), France (13) or Denmark (14) these reports already exist and reveal the current resistance situation. In addition, there is only a small number of national publications concerning this important topic in Austria.

*Pasteurella multocida* (*P. multocida*) can infect a wide variety of animal species. It is an important pathogen in livestock, wildlife and pets, and is also considered as zoonotic agent (15). In cattle and buffalos, it can cause hemorrhagic septicemia, characterized by high rates of morbidity and mortality, mostly seen in Africa and Asia (16) In America and Europe, *P. multocida* is frequently isolated from artiodactyls suffering from pneumoniae. Furthermore, *P. multocida* is known to cause rhinitis atrophicans in pigs, snuffles in rabbits and fowl cholera in poultry (15). Until now, antimicrobial medication is still the first choice in controlling *P. multocida* infections. Over the past decades, antimicrobial resistant strains have emerged, especially in cattle, pigs and poultry, impending successful treatment (17–19). The increasing detection rate of antimicrobial resistant *P. multocida,* along with new Austrian legislation mandating obligatory antibiograms for specific antibiotic applications, has brought antimicrobial susceptibility testing into the focus (20, 21). The newest Clinical and Laboratory Standards Institute (CLSI) document advises to use cation adjusted Mueller Hinton Broth for antimicrobial susceptibility testing of *P. multocida*. If the strains fail to grow, laked horse blood is recommended as a supplement (22). Many members of the family *Pasteurellacae* require an enriched CO_2_ atmosphere for their proper growth, such as *Actinobacillus pleuropneumoniae* (23), *Glaesserella parasuis* (24), *Avibacterium paragallinarum* (25) or the newly identified species *Mannheimia pernigra* (26). Also, for *P. multocida,* an enriched CO_2_ atmosphere is beneficial for its growth (27). The aim of our study was to determine if optimized growth conditions lead to more reliable results in antimicrobial susceptibility testing. To obtain a full comprehension of the resistance patterns, we employed broth microdilution using various methods, along with whole genome sequencing, to ascertain which approach best correlates with detected resistance genes.

## 2 Material and methods

### 2.1 Isolates

We included a total of 276 isolates of *P. multocida* in the study. Of the 276 isolates, 95 were isolated from pigs, 69 from cattle, 30 from cats, 26 from poultry, 26 from rabbits, 7 from dogs, 6 from humans, 5 from small ruminants, 5 from wild boars, 3 from chamois, 2 from deer and one each from a hare and a mouse. The majority (166 isolates) were obtained from our routine veterinary diagnostic work at the Agency for Health and Food Safety (AGES). From 2008 to early 2023 isolated *P. multocida* strains were collected and stored in Proteose Peptone (Oxoid, Hamshire, UK)-Glycerin (Merck, Darmstadt, Germany) solution at −80°C until further use.

In summary, the pathologically altered organs of submitted deceased animals were cut open with a sterile scalpel, and a swab was taken from the inner part of the organ. The swab was then plated onto different agar plates and incubated at 37°C in various atmospheres depending on the expected bacteria’s requirements. Grayish, smooth colonies showing no hemolysis and no growth on MacConkey Agar were subcultured and identified using API® 20 E (Biomérieux, Marcy-l’Étoile, France) or MALDI TOF/ MALDI Biotyper Sirius IVD System, MBT Compass HT Version 5.1.300 (Bruker Daltonics GmbH & Co. KG, Bremen, Germany).

For cattle, swine, rabbits and wildlife, the source of isolation was the lung, except 3 strains which originate from the upper respiratory tract of living pigs. In poultry, septicemia was the predominant clinical presentation, leading to the isolation of *P. multocida* from numerous inner organs such as spleen, liver, lung and heart. If multiple organs were affected, the isolate from the lung was used for further examinations.

To capture a wider spectrum of different isolates, the University of Veterinary Medicine Vienna (VMU) and the Institute for Veterinary Diagnostics in Carinthia (ILV) supported our study by providing isolates. The majority of samples from pet animals including cats, dogs and rabbits were provided by the VMU while the ILV also contributed samples from various hosts.

The human isolates were provided by the Institute of Medical Microbiology and Hygiene (AGES, Vienna). The strains were all related to pet bite incidents in 2023 in Vienna.

All isolates originate from diseased animals in which *P. multocida* was the source of infection or part of a coinfection with other bacterial or viral agents. Samples originated from all regions of Austria except the Federal State of Vorarlberg. In the case where multiple samples originated from the same location, only one isolate per year, per animal, and per farm was considered for analysis.

### 2.2 Phenotypic testing

We analyzed the antimicrobial susceptibility of all *P. multocida* isolates using broth-microdilution. The minimal inhibitory concentration was tested in three different approaches: (1) standard testing for gram negative bacteria with CAMHB (cation adjusted Mueller Hinton Broth, Bruker Daltonics GmbH & Co. KG, Bremen, Germany) (2) CAMHB supplemented with 2.5% of LHB (laked horse blood, Thermo Sientific, Hampshire, UK) as used for fastidious microorganisms (3) CAMHB supplemented with 2.5% of LHB and with 5–10% CO_2_. These mentioned approaches will be referenced in the script as method CAMHB, method LHB and method LHB + CO_2_.

The microdilution was performed using a commercial standard 96 well layout MICRONAUT S Großtiere (Bruker Daltonics GmbH & Co. KG, Bremen, Germany) according to the manufacturers protocol and measured photometrically using the MICRONAUT 6 software (Bruker Daltonics GmbH & Co. KG, Bremen, Germany) on the next day. When the photometric output indicated a longer incubation time or visual check due to cloudy unclear results as was observed for many isolates using only CAMHB, the results were verified manually. The quantitative data was then interpreted using veterinary specific breakpoints for cattle and pigs in reference to the guidelines from CLSI (22). As the enriched CO_2_ atmosphere is not a recommended method by CLSI, the clinical break points can only be used as an orientation. Due to the various species and different isolation origins in the remaining categories, the Minimum Inhibitory Concentration 90 (MIC_90_) was used. MIC_90_ represents the concentration of an antibiotic at which 90% of the tested bacterial population is inhibited (28). The antibiotics tested and their respective dilution ranges are as follows penicillin G (0.0625–2 μg/mL), ampicillin (0.03125–16 μg/mL), amoxicillin/clavulanic acid (1/0.5–16/8 μg/mL), ceftiofur (0.125–4 μg/mL), enrofloxacin (0.0156–1 μg/mL), florfenicol (1–8 μg/mL), colistin (0.5–2 μg/mL), tetracycline (0.25–8 μg/mL), trimethoprim/sulfamethoxazole (0.125/2.375–2/38 μg/mL), erythromycin (2–4 μg/mL), tiamulin (0.25–16 μg/mL), tilmicosin (0.5–16 μg/mL), tildipirosin (1–32 μg/mL), gamithromycin (0.25–8 μg/mL), tulathromycin (1–64 μg/mL), gentamicin (0.0625–8 μg/mL). The reference strains *Staphylococcus aureus* (ATCC 29213), *Escherichia coli* (ATCC 25922), *Pseudomonas aeruginosa* (ATCC 27853) and *Streptococcus pneumoniae* (ATCC 49619) were used as quality controls, as described by CLSI (25).

### 2.3 Genotypic testing

Genomic DNA for Illumina Next Seq2000 (Illumina, San Diego, CA, USA) sequencing was isolated using the MAG Attract HMW (Qiagen, Hilden, Germany) according to the manufacturer’s instruction. DropSense 16 (Trinean NV, Gentbrugge, Belgium) was used to verify DNA purity. Library preparation was performed using Nextera XT DNA Library Preparation Kit (Illumina, San Diego, CA, USA). Paired-end sequencing was performed with a read length of 2 × 150 bp. Raw reads were trimmed using Trimmomatic and *de novo* assembled in SPAdes v3.15.2 (29, 30). All sequences were analyzed using PubMLST: Species ID, to detect possible contaminations (31). Contaminated sequences were excluded from the study. The strains were then screened for antimicrobial resistance genes using the AMRFinderPlus software version:3.11.2 (32). Resistance genes that were identified with “exact,” “blast” or “partial contig end” were included in our study (33).

### 2.4 Statistical analysis

Data was tested for normal distribution by the Kolmogorow-Smirnov-test. Most parameters were not normally distributed, therefore significant differences of MIC values between the growth methods were assessed with the Friedman’s variance analysis test followed by the Wilcoxon test. Descriptive statistics was undertaken to determine the frequency of antimicrobial susceptibility or resistance. Bacterial growth and CLSI data of the groups were compared using the Fisher’s exact test. All data was compiled with Microsoft Excel 21 and analyzed using IBM SPSS statistics (version 29).

## 3 Results

### 3.1 Phenotypic testing

**Figure 1:**
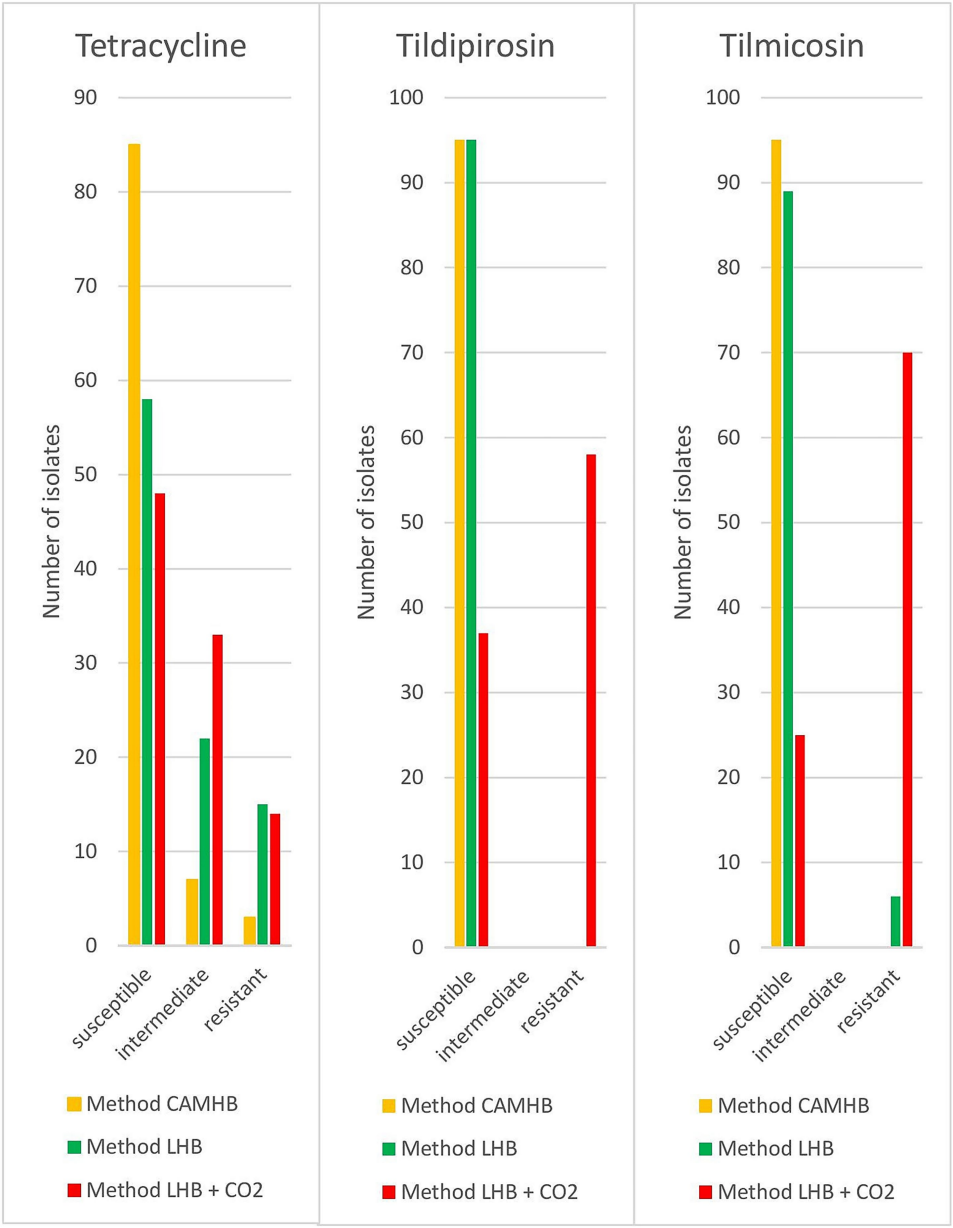
Results of different approaches for resistance testing of porcine *P. multocida* (*n* = 95) isolates.

The antimicrobial susceptibility testing results for the 95 clinical isolates from pigs were evaluated only for CLSI listed antibiotics indicated for the treatment of *P. multocida*. For method CAMHB, 28% of the pig isolates had to be evaluated manually. Isolates tested with method CAMHB indicated 3% resistance to tetracycline, while no other resistance could be detected. Method LHB showed resistance rates of 3% to ampicillin, 14% to tetracycline and 5% to tilmicosin. Resistance detected using method LHB + CO_2_ was as follows: 5% ampicillin, 2% penicillin, 13% tetracycline, 60% tildipirosin and 74% tilmicosin. The results conferring to their various testing methods differed greatly. Tildipirosin and tilmicosin indicated significantly (*p* < 0.05) more resistant strains with method LHB + CO_2_ (Figure 1). Method LHB + CO_2_ identified a resistance rate of 60% for tildipirosin, whereas the CAMHB and LHB methods did not detect any resistant strains. All isolates tested with method CAMHB were susceptible for tilmicosin. Method LHB yielded resistance rates of 5% and method LHB + CO_2_ detected 74% resistant strains. For tetracycline, method LHB and LHB + CO_2_ indicated significantly (*p* < 0.05) more intermediate and resistant isolates than method CAMHB. With method CAMHB, 90% of the isolates were interpreted as susceptible, 7% as intermediate and 3% as resistant. Method LHB showed 61% of susceptible isolates, 25% were intermediate and 14% were resistant. While using method LHB + CO_2_, 51% of isolates were susceptible, 36% were intermediate and 13% were resistant. For ampicillin, penicillin, ceftiofur, florfenicol, tulathromycin and enrofloxacin no significant discrepancies have been found in the evaluation of the different methods.

**Figure 2:**
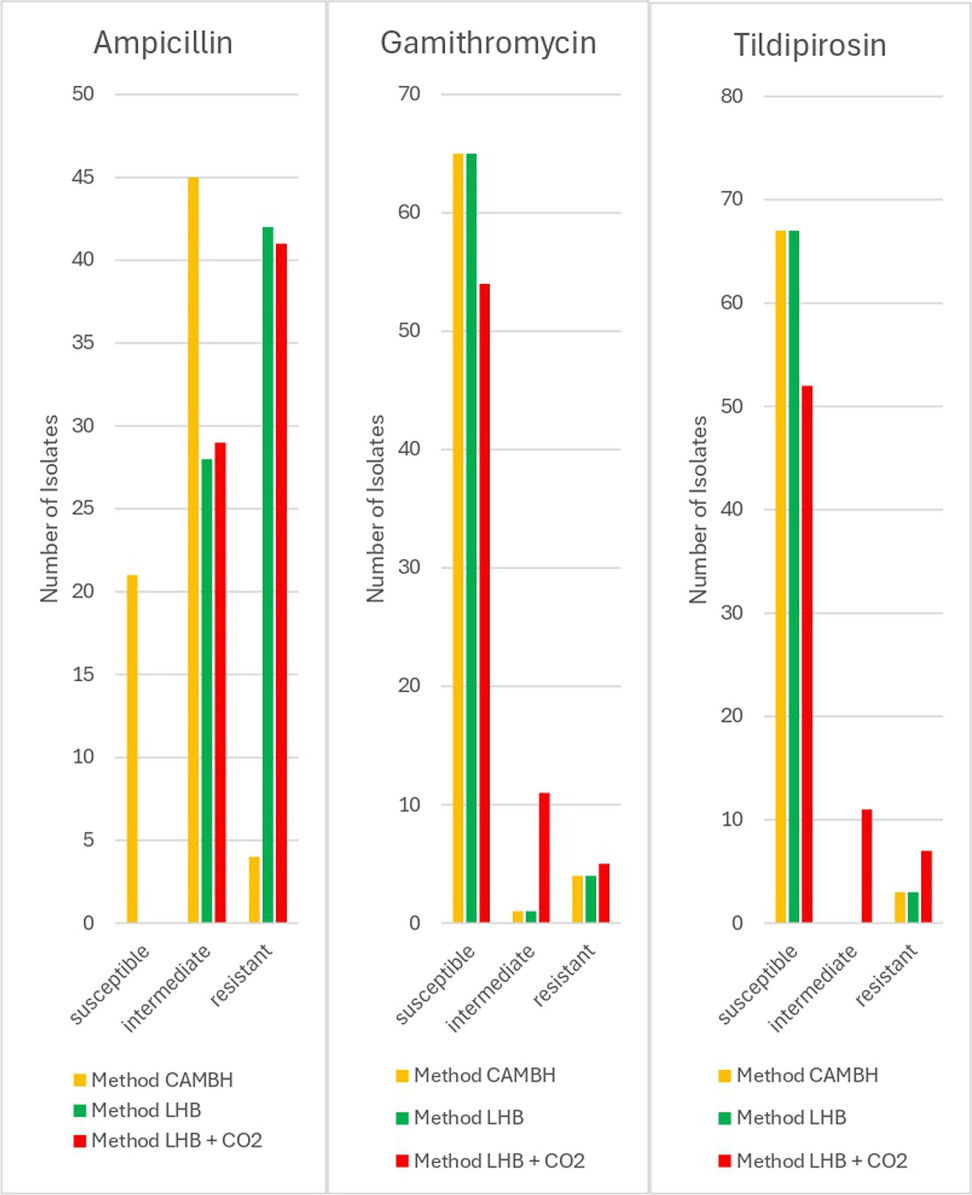
Results of different approaches for resistance testing of bovine *P. multocida* (*n* = 70) isolates.

In cattle, 30% of the isolates tested with method CAMHB had to be verified visually. Bovine isolates tested with method CAMHB showed resistance rates of 7% to ampicillin, 1% to enrofloxacin, 1% to florfenicol, 6% to gamithromycin, 4% to tildipirosin, 13% to tetracycline, and 6% to tulathromycin. Method LHB revealed resistance rates of 61% to ampicillin, 4% to enrofloxacin, 3% to florfenicol, 6% to gamithromycin, 4% to tildipirosin, 20% to tetracycline, and 7% to tulathromycin. Method LHB + CO_2_ indicated resistance rates in 59% to ampicillin, 4% to enrofloxacin, 1% to florfenicol, 7% to gamithromycin, 10% to tildipirosin, 14% to tetracycline, 7% to tulathromycin. The method LHB + CO_2_ resulted in significantly (*p* < 0.05) more intermediate isolates for the macrolides gamithromycin and tildipirosin. For gamithromycin, the results from method CAMBH and method LHB were identical. In total 93% of the tested strains were interpreted as susceptible, 1% as intermediate and 6% as resistant. Method LHB + CO_2_ indicated 77% as susceptible, 16% as intermediate and 7% as resistant isolates. For tildipirosin, methods CAMHB and LHB again produced identical results, identifying 96% of susceptible and 4% of resistant isolates. Method LHB + CO_2_ showed 74% of susceptible strains, 16% of intermediate strains and 10% of resistant strains. In ampicillin, method LHB and LHB + CO_2_ indicated significantly (*p* < 0.05) more resistant isolates. Method CAMHB yielded 30% for susceptible isolates, 64% for intermediate isolates and 6% for resistant isolates. Method LHB identified 40% intermediate strains and 60% resistant strains. Method LHB + CO_2_ led to similar results with 41% of the isolates being identified as intermediate and 59% as resistant isolates (Figure 2). For penicillin, ceftiofur, florfenicol, tulathromycin, tetracycline and enrofloxacin no significant deviations occurred.

A total of 54% of the poultry strains tested with method CAMHB had to be evaluated manually. Most of our data showed a similar output in the different cultivation approaches, with high concentrations obtained for colistin (>2 μg/mL) and tiamulin (>16 μL/mL). Major discrepancies were seen again in macrolides. Four-fold higher MIC_90_ values were obtained for tulathromycin as well as for tildipirosin with method LHB + CO_2_ (Table 1).

**Table 1:** Distribution of MIC-values from poultry (*n* = 26) in different approaches.

Antibiotics	Approach	0.015	0.03	0.06	0.12	0.25	0.5	1	2	4	8	16	32	64	MIC 90
AMC	1)							26							1
	2)							26							1
	3)							26							1
AMP	1)		2	7	16	1									0.12
	2)				11	12	3								0.5
	3)				5	15	4	2							0.5
CET	1)				26										0.12
	2)				17	2	5	2							0.5
	3)				14	3	6	3							1
COL	1)						9	10	7						2
	2)						1	5	20						2
	3)							3	23						2
ENR	1)	20	5	1											0.03
	2)	15	8	2			1								0.06
	3)	12	10	3			1								0.06
ERY	1)								26						2
	2)								15	11					4
	3)								3	23					4
FLL	1)							26							1
	2)							26							1
	3)							26							1
GAM	1)					9	9	8							1
	2)					3	13	8	2						1
	3)						3	3	10	10					4
GEN	1)			2		1	11	12							1
	2)						1	16	9						2
	3)							3	12	11					4
PEN	1)			24	2										0.06
	2)			6	17	3									0.25
	3)			9	16				1						0.12
T/S	1)				25	1									0.12
	2)				25			1							0.12
	3)				23	2			1						0.25
TDP	1)							26							1
	2)							23	3						2
	3)							2	3	4	14	3			16
TET	1)					25	1								0.25
	2)					7	14	5							1
	3)					8	14	3	1						1
TIA	1)					2				2	9	13			16
	2)											26			16
	3)										1	25			16
TILM	1)						2		5	19					4
	2)								1	10	13	2			8
	3)									1	7	18			16
TUL	1)							25	1						1
	2)							17	9						2
	3)							2	1	4	9	10			16

1) With Müller Hinton Broth, 2) supplemented with 2.5% blood, 3) supplemented with 2.5% blood and enriched CO_2_ atmosphere. The ranges of tested antibiotics are shaded. The concentration which inhibits 90% of the bacteria is highlighted in green = MIC _90_.

AMC, Amoxicilin/Clavulanic acid; AMP, Ampicillin; CET, Ceftiofur; COL, Colistin; ENR, Enrofloxacin; ERY, Erythromycin; FLL, Florfenicol; GAM, Gamithromycin; GEN, Gentamicin; PEN, Penicillin; T/S, Trimethoprim/Sulfamethoxazole; TDP, Tildipirosin; TET, Tetracycline, TIA, Tiamulin; TILM, Tilmicosin; TUL, Tulathromycin.

The color shaded ares should remain shaded since it outlines the tested range of the respective antibiotic.

Cats, dogs, rabbits and a mouse were analyzed together under the pets category because they originate from private households. In general, the minimal inhibition concentration was found to be in the lower half for most of the antibiotics. Using method CAMHB, 55% of analyzed pet isolates had to be assessed visually. As seen in poultry, minimal inhibiting concentrations were high in colistin (1 μg/mL) and tiamulin (>16 μg/mL). Once again macrolides showed substantial differences when tested usingLHB+CO_2_ method, with up to five-fold higher values for tulathromycin and tildipirosin compared to the unsupplemented incubation method (Table 2).

**Table 2:** Distribution of MIC-values from pets (*n* = 64) in different approaches.

Antibiotics	Approach	0.015	0.03	0.06	0.12	0.25	0.5	1	2	4	8	16	32	64	MIC 90
AMC	1)							64							1
	2)							64							1
	3)							64							1
AMP	1)		17	24	23										0.12
	2)		2	2	29	25	5	1							0.25
	3)		1	4	25	28	5			1					0.25
CET	1)				64										0.12
	2)				52	5	3	3	1						0.5
	3)				47	10	5	2							0.5
COL	1)						55	7	2						1
	2)						10	20	34						2
	3)						4	3	57						2
ENR	1)	54	6	4											0.03
	2)	28	26	4	6										0.06
	3)	34	20	5	4	1									0.06
ERY	1)								63	1					2
	2)								42	22					4
	3)								14	50					4
FLL	1)							64							1
	2)							64							1
	3)							63	1						1
GAM	1)					21	27	16							1
	2)					15	21	25	2	1					1
	3)					3	7	16	21	10	7				4
GEN	1)			14	6	8	20	16							1
	2)			3	1	9	7	33	10	1					2
	3)			1	2	6		15	32	7	1				4
PEN	1)			63	1										0.06
	2)			29	26	9									0.25
	3)			37	21	5	1								0.12
T/S	1)				64										0.12
	2)				64										0.12
	3)				61	3									0.12
TDP	1)							64							1
	2)							55	8	1					2
	3)							14	5	21	16	7	1		16
TET	1)					51	12	1							0.5
	2)					22	25	13	4						1
	3)					25	21	10	1	7					4
TIA	1)					14		1	4	12	17	16			16
	2)					2		1		2	5	54			16
	3)					3		1		2	1	57			16
TILM	1)						19	0	21	23	1				4
	2)						4		8	18	23	11			16
	3)						3	1	2	7	12	39			16
TUL	1)							62	2						1
	2)							58	5	1					1
	3)							13	6	10	17	17		1	16

1) With Müller Hinton Broth, 2) supplemented with 2.5% blood, 3) supplemented with 2.5% blood and enriched CO_2_ atmosphere. The ranges of tested antibiotics are shaded. The concentration which inhibits 90% of the bacteria is highlighted in green = MIC _90_.

AMC, Amoxicilin/Clavulanic acid; AMP, Ampicillin; CET, Ceftiofur; COL, Colistin; ENR, Enrofloxacin; ERY, Erythromycin; FLL, Florfenicol; GAM, Gamithromycin; GEN, Gentamicin; PEN, Penicillin; T/S, Trimethoprim/Sulfamethoxazole; TDP, Tildipirosin; TET, Tetracycline, TIA, Tiamulin; TILM, Tilmicosin; TUL, Tulathromycin.

The color shaded ares should remain shaded since it outlines the tested range of the respective antibiotic.

Due to the small number of isolates from wildlife (*n* = 11), small ruminants (*n* = 5) and human isolates (*n* = 6) the graphical presentations have been waived. By method CAMHB, 45% of the tested strains of this species group had to be verified manually. These host categories demonstrated MIC-values situated in the lower ranges of the antibiotic dilutions for the method CAMHB. Method LHB and LHB + CO_2_ resulted in higher MIC values in macrolides, similar to the previous data from other animal classes.

### 3.2 Genotypic testing

In our study, 25 of 276 isolates had at least one resistance gene. Of these strains, 15 strains originated from cattle and the remaining 10 isolates were obtained from swine. None of the other specimens showed any kind of resistance genes.

In cattle samples, 20% carried resistance genes against tetracyclines (*tet(H)*, *tet(Y)*). Followed by 19% carrying resistance genes against streptomycin (*aph(3″)-Ib, aph(6)-Ib*), 17% against sulfonamides (*sul2*), 16% against kanamycin (*aph(3′)-Ia*),7% against chloramphenicol (*catA3*), 6% against macrolides (*mef(C)*, *mph(G)*) and streptomycin/spectinomycin (*aadA1*, *aadA31*), 3% against gentamicin (*aac(3)-lle*) and 2% against florfenicol (*floR*). In total 10% of the tested isolates were carrying three or more resistance genes against different antibiotic classes.

The most common resistance gene detected in isolates from swine was *sul2 in* 7% followed by genes conferring resistance to streptomycin (*aph(3″)-Ib, aph(6)-Id*) in 5% and the trimethoprim insensitive dihydrofolate reductase encoded by *dfrA14* and *dfrA1* (34) in 3%. Resistance genes against tetracyclines (*tet(B), tet(H)*) were found in 2% of the tested isolates. Only 1% was carrying a resistance gene against kanamycin (*aph(3′)-Ia*) and lincosamides (*lnu(F)*). Three isolates from pigs harbored 3 resistance genes against various antibiotics classes. Further information about the quality of the found resistance genes in our isolates are available at the Supplementary Table 1.

### 3.3 Comparison between phenotype and genotype

In isolates from pigs using method CAMHB, ceftiofur, florfenicol, penicillin, tildipirosin, tilmicosin and tulathromycin demonstrated perfect concordance between phenotypic and genotypic resistance profiles. Discrepancies were only detected in tetracycline which resulted in a total concordance of 99.6%. In two phenotypical resistant isolates no corresponding genotype could be detected. One isolate carrying *tet(B)* was interpreted as intermediate (Table 3).

**Table 3:** Comparison of phenotypical resistance to genotypical resistance from porcine *P. multocida* (*n* = 95) using Cation adjusted Mueller Hinton Bouillion.

Antibiotics	P+/G+	P−/G−	P+/G−	P−/G+	Concordance
AMP	0	95	0	0	100%
CEF	0	95	0	0	100%
FFL	0	95	0	0	100%
PEN	0	95	0	0	100%
TET	1	91	2	1	96.84%
TDP	0	95	0	0	100%
TILM	0	95	0	0	100%
TUL	0	95	0	0	100%
Concordance total					99.6%

AMP, Ampicillin; CET, Ceftiofur; FLL, Florfenicol; PEN, Penicillin; TET, Tetracycline; TDP, Tildipirosin; TILM, Tilmicosin; TUL, Tulathromycin; P+, resistant phenotype; P−, non-resistant phenotype; G+, resistant genotype; G−, non-resistant genotype.

With the supplementation of LHB the total concordance rate decreased to 97.6%. Ceftiofur, florfenicol, penicillin, tildipirosin and tulathromycin again showed a perfect concordance. Both strains carrying a resistance gene against tetracycline were detected correctly by microdilution. In 11 samples, only a phenotypical resistance against tetracycline could be detected. In two phenotypical ampicillin resistant isolates, no responsible resistance gene could be found. Against tilmicosin five isolates showed a resistant phenotype without a matching resistance gene (Table 4).

**Table 4:** Comparison of phenotypical resistance to genotypical resistance from porcine *P. multocida* (*n* = 95) using Cation adjusted Mueller Hinton Bouillion supplemented with 2.5% lysed horse blood.

Antibiotics	P+/G+	P−/G−	P+/G−	P−/G+	Concordance
AMP	0	93	2	0	97.90%
CEF	0	95	0	0	100%
FFL	0	95	0	0	100%
PEN	0	95	0	0	100%
TET	2	82	11	0	88.42%
TDP	0	95	0	0	100%
TILM	0	90	5	0	94.74%
TUL	0	95	0	0	100%
Concordance total					97.6%

AMP, Ampicillin; CET, Ceftiofur; FLL, Florfenicol; PEN, Penicillin; TET, Tetracycline; TDP, Tildipirosin; TILM, Tilmicosin; TUL, Tulathromycin; P+, resistant phenotype; P−, non-resistant phenotype; G+, resistant genotype; G−, non-resistant genotype.

Porcine strains incubated in an enriched CO_2_ atmosphere resulted in the overall lowest concordance rate of 81.1%. Only ceftiofur, florfenicol and tulathromycin showed no differences in phenotype and genotype. As seen with the blood supplementation method, the tetracycline resistant strains could be correctly detected by AST. In 10 isolates, only a phenotypical resistance could be detected. Four strains for ampicillin and two strains for penicillin were interpreted as a resistant phenotype with no matching resistance gene. The highest numbers of phenotypical resistant isolates, without a respective genotype, were seen in macrolides such as tildipirosin and tilmicosin. In tildipirosin 57 isolates were phenotypically interpreted as resistant and in tilmicosin 70 isolates were without a corresponding resistance gene (Table 5).

**Table 5:** Comparison of phenotypical resistance to genotypical resistance from porcine *P. multocida* (*n* = 95) using Cation adjusted Mueller Hinton Bouillion supplemented with 2.5% lysed horse blood and enriched CO_2_ atmosphere.

Antibiotics	P+/G+	P−/G−	P+/G−	P−/G+	Concordance
AMP	0	91	4	0	95.79%
CEF	0	95	0	0	100%
FFL	0	95	0	0	100%
PEN	0	93	2	0	97.90%
TET	2	83	10	0	89.47%
TDP	0	38	57	0	40%
TILM	0	25	70	0	26.13%
TUL	0	95	0	0	100%
Concordance total					81.1%

AMP, Ampicillin; CET, Ceftiofur; FLL, Florfenicol; PEN, Penicillin; TET, Tetracycline; TDP, Tildipirosin; TILM, Tilmicosin; TUL, Tulathromycin; P+, resistant phenotype; P−, non-resistant phenotype; G+, resistant genotype; G−, non-resistant genotype.

Strains isolated from cattle tested for their antimicrobial resistance via microdilution using CAMHB showed a concordance score of 96.9%. Ceftiofur, penicillin and gamithromycin had perfect concordance. For tetracycline, eight isolates showed a phenotypical resistance as well as a resistant genotype. For six isolates containing a resistance gene, the phenotypical resistance could not be ascertained. Therefore, one phenotypical resistant strain did not show the corresponding genotype. For 63 strains, an unanimous pheno- and genotype in ampicillin resulted in a concordance rate of 90%. Four ampicillin resistant phenotypes could not be confirmed by whole genome sequencing. The macrolides tildipirosin and tulathromycin had one phenotypical resistant isolate without a matching genotype. Furthermore, two strains having a macrolide resistance gene could not be confirmed by microdilution in the case of tildipirosin and one strain regarding to tulathromycin. Of the two strains carrying *floR*, one resulted in a resistant phenotype, the other was considered susceptible (Table 6).

**Table 6:** Comparison of phenotypical resistance to genotypical resistance from bovine *P. multocida* (*n* = 69) using Cation adjusted Mueller Hinton Bouillion.

Antibiotics	P+/G+	P−/G−	P+/G−	P−/G+	Concordance
AMP	0	65	4	0	94.20%
CEF	0	69	0	0	100%
FFL	1	67	0	1	98.55%
PEN	0	69	0	0	100%
TET	8	54	1	6	89.86%
TDP	2	64	1	2	95.65%
GAM	4	65	0	0	100%
TUL	3	64	1	1	97.10%
Concordance total					96.92%

AMP, Ampicillin; CET, Ceftiofur; FLL, Florfenicol; PEN, Penicillin; GAM, Gamithromycin; TET, Tetracycline; TDP, Tildipirosin; TUL, Tulathromycin; P+, resistant phenotype, P−, non-resistant phenotype; G+, resistant genotype, G−, non-resistant genotype.

As seen in porcine isolates, the total concordance rate decreased to 91.3% with the supplementation of LHB. This was mainly caused by the increase of ampicillin resistant phenotypes without a responsible resistance gene, seen in 42 isolates. The remaining antibiotics showed an equal or even higher concordance rate compared to the CAMHB approach. A perfect concordance was achieved in ceftiofur, florfenicol, penicillin and gamithromycin. Tildipirosin showed the same results as method CAMBH. Eleven isolates containing a tetracycline resistance gene presented a phenotypical resistance. Only one strain with a resistance gene was not detected by AST and one resistant phenotype showed no resistant genotype. In tulathromycin all the isolates carrying a resistance gene were identified as phenotypically resistant. One strain showed only a phenotypical resistance type (Table 7).

**Table 7:** Comparison of phenotypical resistance to genotypical resistance from bovine *P. multocida* (*n* = 69) using Cation adjusted Mueller Hinton Bouillion supplemented with 2.5% lysed horse blood.

Antibiotics	P+/G+	P−/G−	P+/G−	P−/G+	Concordance
AMP	0	27	42	0	39.13%
CEF	0	69	0	0	100%
FFL	2	67	0	0	100%
PEN	0	69	0	0	100%
TET	13	54	1	1	97.10%
TDP	2	64	1	2	95.65%
GAM	4	65	0	0	100%
TUL	4	64	1	0	98.55%
Concordance total					91.30%

AMP, Ampicillin; CET, Ceftiofur; FLL, Florfenicol; PEN, Penicillin; GAM, Gamithromycin; TET, Tetracycline; TDP, Tildipirosin; TUL, Tulathromycin; P+, resistant phenotype, P−, non-resistant phenotype; G+, resistant genotype, G−, non-resistant genotype.

Cattle isolates with an enriched CO_2_ environment resulted in a concordance rate of 90%. Only ceftiofur and penicillin showed a perfect concordance. For ampicillin, 41 isolates were interpreted as resistant without a responsible resistance gene. Five isolates containing a tetracycline resistant gene did not show a resistant phenotype whereas one phenotypical resistant strain was detected without a resistance gene. Four isolates presented a phenotypical tildipirosin resistance with no corresponding genotype. One strain carrying a resistance gene was not considered resistant by microdilution. Of the two florfenicol resistant genotypes only one resulted in a phenotypical resistance. In gamithromycin and tulathromycin all four isolates containing a macrolide resistance gene also showed a phenotypical resistance. One strain was interpreted as resistant in both antibiotics, but a genomic explanation could not be found (Table 8).

**Table 8:** Comparison of phenotypical resistance to genotypical resistance from bovine *P. multocida* (*n* = 69) using Cation adjusted Mueller Hinton Bouillion supplemented with 2.5% lysed horse blood and enriched CO_2_ atmosphere.

Antibiotics	P+/G+	P−/G−	P+/G−	P−/G+	Concordance
AMP	0	28	41	0	40.58%
CEF	0	69	0	0	100%
FFL	1	67	0	1	98.1%
PEN	0	69	0	0	100%
TET	9	54	1	5	91.3%
TDP	3	61	4	1	92.75%
GAM	4	64	1	0	98.55%
TUL	4	64	1	0	98.55%
Concordance total					90%

AMP, Ampicillin; CET, Ceftiofur; FLL, Florfenicol; PEN, Penicillin; GAM, Gamithromycin; TET, Tetracycline; TDP, Tildipirosin; TUL, Tulathromycin; P+, resistant phenotype, P−, non-resistant phenotype; G+, resistant genotype, G−, non-resistant genotype.

## 4 Discussion

To our knowledge, this is the first study on antimicrobial resistance of *P. multocida* in Austria, and the first to examine the impact of different incubation methods on the phenotypic resistance testing outcome in *P. multocida*.

In the past decades, there have been numerous studies on resistance in *P. multocida* worldwide. However, information on the exact method of susceptibility testing is barely described. Most researchers rely on the CLSI-documents, but whether they supplement with LHB or not during their examinations often remains unknown. Overall, with method CAMBH, every third sample did not demonstrate sufficient growth and the method LHB + CO_2_ is not a prescribed testing method. Therefore, in the following discussion our results from method LHB will be compared with the established literature.

In comparison to other European countries the resistance level for porcine *P. multocida* isolates in Austria is still low (10, 12, 35, 36). In a Czech study with 332 porcine isolates the resistance rate for Tetracycline was over 30% (36). In Spain resistance in Tetracycline is similar to our findings but they are dealing with a much higher resistance rate against beta lactams of about 40% (35). Our study differs greatly from Chinese findings, which report high resistance rates for ampicillin, tetracycline and trimethoprim-sulfamethoxazole (37).

Regarding bovine isolates, Germany’s resistance rates for ampicillin with 88%, tetracycline with 49% and tulathromycin with 21% were much higher whereas resistance against florfenicol and enrofloxacin were similar low as in our study. The results of our study differ greatly from the published data from the United States of America displaying resistance rates much higher than 50% to tetracycline, tilmicosin, tildipirosin, gamithromycin, enrofloxacin and florfenicol (38).

For *P. multocida* infections in poultry no interpretative criteria for susceptibility testing are available. In Austria more than 50% of the antibiotics sold for poultry belong to broad spectrum penicillin and macrolides represented by ampicillin and tilmicosin in our microdilution layout (39). Nevertheless, no elevated MICs could be detected for these antibiotic classes (see Table 3). A recent published study from the neighboring country Hungary on *P. multocida* in waterfowl showed comparable results in colistin and tiamulin. In contrast to our study more than 40% of the Hungarian isolates showed MIC ≥ 0.5 μg/mL for enrofloxacin whereas we detected just one single isolate reaching that value (40). Hints of multi drug resistance in avian *P. multocida* as seen in Asian and African studies could not be detected (41–44).

In pets no veterinary specific breakpoints were applied, so a precise forecast of the success of an antimicrobial therapy cannot be made. Depending on the low MIC-values for commonly used antibiotics like penicillins, aminopenicillins, cephalosporins and fluoroquinolones we suspect good efficacy. The method LHB and method LHB + CO_2_ mainly resulted in a two- or three-fold higher MIC_90_ compared to method CAMHB. Very few studies examine MIC via broth microdilution on pet samples. Agar diffusion testing is commonly used with unequal interpretative criteria. This divers AST complicates meaningful comprehension. As seen in other European studies of *P. multocida* in cats and dogs, the resistance level is very low (45, 46). In Brazil, isolates from cats, dogs and rabbits showed increased resistance rates for sulphonamides and the combined drug trimethoprim-sulfamethoxazole (47). In our study no strain from the pet category exceeded the first dilution step of 0.125/2.375 μg/mL for trimethoprim-sulfamethoxazole, probably indicating susceptibility. A comparably high-level resistance rate in beta lactams in cat isolates was detected in Iran (48). This data is in strong contrast to our low MIC 90 of 0.25 μg/mL for ampicillin and penicillin. Resistance in rabbit isolates has rarely been detected but Asian studies indicate a moderate resistance to aminoglycosides (49, 50).

Strains from humans, small ruminants and wildlife showed overall low MIC values in the method CAMBH and method LHB. Method LHB + CO_2_ resulted in slightly higher MICs for Tiamulin and Tilmicosin. Nevertheless, our data should be interpreted with caution due to the small number of tested isolates. Information on resistance data of *P. multocida* of these included hosts is very limited. A retrospective study on human cases concluded a good efficacy for tetracycline, chloramphenicol, carbapenems, quinolones, penicillins and cephalosporins which concurs with our results. The mentioned high resistance rates for macrolides could not be verified in Austria (51). Our study confirms the postulated low resistance in small ruminants by Ujvàri and Magyar, 2022 (52). High resistance levels against beta lactams, as seen in India could not be detected (53). Although our study did not reveal suspicious MIC values in wildlife, Spanish colleagues found a phenotypical multi resistant *P. multocida* from chamois in the Alpine ecosystem (54).

The occurrence of resistance associated genes of Austrian *P. multocida* isolates from pigs is low with only one isolate considered as multi drug resistant, conferring resistance against tetracycline, sulfonamide and multiple agents from the class of aminoglycosides. The most frequent resistance genes were *sul(2)* and *aph(3″)-Ib* which seems to be common among porcine isolates (20). Resistance against tetracycline could only be found in two isolates. The genes mediating resistance were *tet(B)* and *tet(H)* which have been previously detected in porcine isolates from Spain and Vietnam (18, 55). Lincosamide nucleotidyltransferase (lnu) have been found mostly in gram positive bacteria like *Enterococcus* spp., *Staphylococcus* spp. or *Streptococcus* spp. (56, 57). To the author’s knowledge, this is the first case of detecting *lnu(F)* in *P. multocida*, which has previously been reported in *Enterobacterales* (58).

Antimicrobial resistance was seen more often in bovine isolates and furthermore against more antibiotic classes. Genes conferring resistance to tetracyclines *tet(H)* and *tet(Y)* were the most dominant group which could also be confirmed by a study from Japanese colleagues (59). Recent studies about resistance in *Pasteurellacae* indicate that macrolide resistance seems to slowly establish in the cattle population (60–62). The first resistant isolates in Austria were detected in 2022. In contrast to the publications mentioned, we could not detect any beta lactam resistance genes.

The highest concordance rate in cattle and pigs was achieved by method CAMBH. Nevertheless, we do not conclude that this method is the most suitable for susceptibility testing. The poor growth harbors the danger of misinterpreting bacteria as susceptible even when they are carrying resistance genes. Such false negative results can have a negative impact on animal health and welfare as well as financial losses (3). Due to the lack of genetic resistance in porcine isolates, just one tetracycline resistant strain failed to be detected with method CAMHB. Although method CAMHB again led to the highest concordance rate for cattle isolates, resistant isolates could not be detected consistently for florfenicol, tetracycline and tulathromycin. Independent from the animal species, method LHB + CO_2_ led to lowest concordance rate of all, resulting in resistant phenotypes, especially for macrolides, without a genetic explanation. Therefore, it highlights the importance of adhering closely to recommended methods without deviations, but it also raises the question whether LHB in combination with a higher CO_2_ level led to unspecific reactions which can result in a turbidity which in turn can possibly be misinterpreted as bacterial growth (63). Pitfalls for a low concordance of genotype and phenotype could also lie in the genetic analysis. Neither has the responsible gene not been detected or the quality of the sequenced strains is insufficient (64). Since only short-read data and one reference database were used, the absence of a known resistance gene does not exclude the presence of divergent or novel resistance mechanisms. In contrast to fluoroquinolone resistance-mediating mutations, other resistance mediating mutations such as for macrolides and spectinomycin have not been investigated in this study (65). It would be interesting to validate if false positive results also appear in other veterinary pathogens which require blood and CO_2_ such as *Actinobacillus pleuropneumoniae*, *Glaesserella parasuis* or *Trueperella* spp. (22).

However, in bovine strains method LHB would have reached an excellent concordance rate if there would not have been the increase of ampicillin resistant phenotypes. Depenbrock et al. (38) also reported a poor concordance between phenotype and genotype in beta lactams in bovine *P. multocida* strains. Interestingly they detected the resistance gene with a susceptibly phenotype, whereas in our study we encountered the reverse of this problem. It is remarkable that not a single isolate showed a resistance against penicillin which is an anomalous occurrence because both antibiotics belong to beta lactams and share the same resistance mechanism (66). This phenomenon can also be seen in the newest report of GERM-Vet (10) and a study from Switzerland (67) which emphasizes the call for a re-evaluation of clinical breakpoints (68).

Data for enrofloxacin were not shown in the results because their genetic resistance is not covered by the AMRFinder Plus. Fluoroquinolones target the inhibition of enzymes responsible for supercoiling in bacteria which finally leads to cell death. Due to amino acid substitution in the quinolone-resistance-determining-region (QRDR) the antibiotic cannot bind anymore, and the bacteria becomes resistant (69) Three of our 278 tested isolates showed a MIC > 1 μg/mL for Enrofloxacin. Variant calling was performed using iVar (70) on the samples. These samples showed the snp Glu84 to Lys or Ser80 to Leu in *parC* (topoisomerase IV) as described elsewhere (71, 72). We randomly selected 10 isolates with MIC of ≤0.0156 μg/mL as negative controls. All 10 samples did not demonstrate any mutations.

Spectinomycin offers veterinary specific breakpoints for *P. multocida* from bovine respiratory diseases. Our Micronaut layout is designed by the recommendations from “Deutsche Veterinärmedizinische Gesellschaft: Arbeitskreis Antibiotikaresistenzen” (73) and no longer provides Spectinomycin values. This decision is based on the fact, that there are no single preparations available on German (74) or Austrian (75) markets. Fortunately, we have data from previous microdilution testing with Spectinomycin tested by CAMHB. From 46 isolates tested 33 strains resulted in a non-resistant genotype and phenotype. Both strains containing a resistance gene were phenotypically interpreted as resistant. Eleven isolates showed a phenotypical resistance without a corresponding genotype. This leads to a concordance rate of 76.08%. In gram negative bacteria like *P. multocida* resistance against spectinomycin is mostly contributed by aminoglycoside adenylyltransferases (76). Interestingly all three bovine strains (one without phenotypically spectinomycin susceptibility testing) were carrying the relatively new discovered *aadA31* resistance gene (77).

Resistant isolates were most frequently isolated from cattle and pigs and were absent in the other animal categories and human cases. In relation to the results from cattle and pigs these findings are similar to studies other European countries like Germany, France and Italy (78, 79). For most of the tested isolates practitioners would have enough options for successful antibiotic therapy. Nevertheless, some resistance profiles of *P. multocida* are concerning. Six strains isolated from cattle plus three isolates from pigs were harboring at least 3 different resistance genes against diverse antibiotic classes which indicate multi drug resistance (80).

## 5 Conclusion

The presence of multi-drug-resistant *P. multocida* in animals highlights the importance of such surveillance studies. A national surveillance program for AMR in veterinary pathogens would be an appropriate tool to notify changes in resistance patterns and furthermore could be utilized by practitioners to support decisions for antimicrobial therapy. More studies should focus on the interaction between phenotype and genotype to provide valuable insight into resistance of pathogenic bacteria. Furthermore, the adoption of new or revised clinical break points should be emphasized. Referring to our study, we would recommend using method LHB for antimicrobial susceptibility testing of *P. multocida.* Method CAMHB often presented uncertain results due to weak growth, which increases the risk of interpreting bacteria as falsely susceptible. Such misinterpretations can cause serious consequences for animal health and welfare. Therefore, we would advise against an enriched CO_2_ atmosphere during the incubation, because in cattle more resistance genes remained undetected and in pigs unspecified turbidity demonstrated falsely resistance.

## Data Availability

The datasets presented in this study can be found in online repositories. The names of the repository/repositories and accession number(s) can be found at http://www.ncbi.nlm.nih.gov/bioproject/1298543, BioProject ID: PRJNA1298543.
